# Dual-Mode Textile Sensor Based on PEDOT:PSS/SWCNTs Composites for Pressure–Temperature Detection

**DOI:** 10.3390/mi16010092

**Published:** 2025-01-14

**Authors:** Ying Wang, Qingchao Zhang, Zhidong Zhang

**Affiliations:** 1 School of Energy and Power Engineering, North University of China, Taiyuan 030051, China; 2 School of Precision Instrument and Optoelectronics Engineering, Tianjin University, 92 Weijin Road, Tianjin 300072, China; zhang_qc_work@163.com; 3 School of Instrument and Electronics, North University of China, Taiyuan 030051, China

**Keywords:** flexible sensor, pressure–temperature sensing, PEDOT:PSS/SWCNTs, e-textiles

## Abstract

As an innovative branch of electronics, intelligent electronic textiles (e-textiles) have broad prospects in applications such as e-skin, human–computer interaction, and smart homes. However, it is still a challenge to distinguish multiple stimuli in the same e-textile. Herein, we propose a dual-parameter smart e-textile that can detect human pulse and body temperature in real time, with high performance and no signal interference. The doping of SWCNTs in PEDOT:PSS improves the electrical conductivity and Seebeck coefficient of the prepared composites, which results in excellent pressure and temperature-sensing properties of the PEDOT:PSS/SWCNTs/CS@PET-textile (PSCP) sensor. The dual-mode sensor has high sensitivity (32.4 kPa^−1^), fast response time (~21 ms), and excellent durability (>2000 times) in pressure detection. Concurrently, this sensor maintains a high Seebeck coefficient of 25 μV/K in the 0–120 K temperature range with a tremendous linear relationship. Based on impressive dual-mode sensing characteristics and independent temperature-difference- and pressure-sensing mechanisms, smart e-textile sensors realize the real-time simultaneous monitoring of weak pulse signals and human body temperature, showing great potential in medical healthcare. In addition, the potential energy is excited by the temperature gradient between the human skin and the environment, which provides a novel idea for wearable self-powered devices.

## 1. Introduction

To simulate the tactile ability of various types of physical stimuli (pressure, strain [1,2,3], temperature [4,5], humidity [6,7], etc.), various piezoresistive [8,9,10], piezoelectric [11,12,13], capacitive [14,15,16], or iontronic [17,18] tactile sensors have been developed to convert external mechanical and thermal effects into detectable electrical signals and to construct independently stretchable multimodal sensing platforms for physiological condition monitoring and body movement detection. Instead of assembling multiple single-function sensors to achieve multi-stimulus detection, it is better to use a single active material to create a multi-functional sensing device, which is a challenging and promising method to meet the needs of high integration and miniaturization [19,20]. Using composite materials with thermoelectric and piezoresistive properties and introducing innovative structural design, a dual-modal sensor that can simultaneously detect temperature and pressure stimuli without interference has been developed [21,22,23,24]. To achieve effective temperature–pressure parameter sensing, ideal thermoelectric materials should have a high Seebeck coefficient, low thermal conductivity, high conductivity, and well-designed structural features. Many thermoelectric materials with high Seebeck coefficients, such as carbon materials and conductive polymers, have been developed for temperature and pressure dual-mode sensing [25,26,27]. In recent years, the combination of organic and inorganic materials has proven to be an effective way to further improve the conductivity and thermoelectric properties of polymers [28,29,30]. Poly (3,4–ethylenedioxythiophene)–poly (styrenesulfonate) (PEDOT:PSS) has been regarded as one of the most promising flexible composites due to its superior thermoelectric properties and highly tunable electrical conductivity. Single-walled CNTs (SWNTs) are renowned for their extremely stable 1D nanostructure and excellent electric and mechanical properties. The SWCNTs also possess a higher Seebeck coefficient, which enhances the integrated thermoelectric properties and electrical conductivity of the composite.

Compared with planar electronic devices, textile electronic devices have the advantages of good air permeability and comfort, easy programming, extensive area bonding, and high robustness. More and more researchers have combined textiles with various composite materials to prepare efficient flexible electronic devices for wearable tactile sensing and health monitoring equipment [31,32,33]. Temperature and pulse are the most common stimuli reflecting body function information and play an indispensable role in preventing, diagnosing, and treating various diseases such as diabetes and cardiovascular diseases. Since body temperature can affect the pulse, flexible sensors that simultaneously obtain temperature and pulse curves provide more accurate and valuable physical health information than sensors that can only detect one stimulus [34,35,36,37]. Wu et al. [38] reported a dual-sensing aerogel that was fabricated with independent pyro-piezoresistive behavior by leveraging MXene [39] and semicrystalline polymer for human physiological monitoring. Therefore, it is of great significance to study multimodal sensors that can effectively discriminate and perceive multiple stimuli for intelligent health monitoring systems [40,41,42,43,44].

In this paper, we propose an economical and efficient strategy to use PEDOT:PSS and SWCNTs composites as active materials for preparing temperature–pressure dual-mode sensors. The 3D spacer fabric structure with high elasticity, flexibility, and thermal insulation is constructed by chitosan-activated PET fibers. Then, the CS@PET textile fibers are treated by vacuum-assisted impregnation with the mixed PEDOT:PSS and SWCNTs solution. The obtained e-textiles have good thermoelectric and piezoresistive effects, which can simultaneously detect temperature and pressure stimuli and convert them into independent voltage and resistance signals, respectively, showing accurate dual-mode sensing without crosstalk. The dual-model sensor has a wide response range, good linear relationship, high sensitivity, fast response, and excellent temperature and pressure response cycle stability. The sensing mechanism was verified by finite element analysis and experiments. The conductive network composed of PEDOT:PSS and SWCNTs and the heat-insulating and elastic fabric structure play a vital role in achieving stable sensing performance. Therefore, this work demonstrates the great potential of intelligent e-textiles in wearable health monitoring devices.

## 2. Experiment Section

### 2.1. Materials

Single-walled carbon nanotubes (SWCNTs) (outer diameter: 1–2 nm, length: 5–30 μm) were purchased from Jiangsu Xianfeng Nanomaterials Science and Technology Co., Ltd. (Nanjing, China). PEDOT:PSS aqueous dispersion (1.5% in water, PH 2.42), chitosan (CS) (medium molecular weight), and glacial acetic acid (concentration: 80%) were purchased from Shanghai Aladdin Biochemistry Science and Technology Co., Ltd. (Aladdin, Shanghai, China). PET spacer textiles were purchased from Aitao Trading Co., Ltd. (Changzhou, China). Silver paste (fineness ≤ 8 μm, viscosity 80–220 Pa.s) was purchased from Shenzhen Saiya Electronic Paste Co., Ltd. (Shenzhen, China).

### 2.2. Fabrication of PEDOT:PSS/SWCNTs/CS@PET Textile

First, 30 mg of SWCNTs and 1 mL of PEDOT:PSS solution were added to the beaker, such that the ratio of carbon nanotubes to PEDOT:PSS was 2:1. Then, 3 mL of deionized water was added and diluted. The mixed solution was ultrasonically treated in a cell grinder for 1 min to make the two mixed evenly. Then, 150 mg of chitosan particles was placed in a beaker containing 50 mL of deionized water, and 0.1 mL of glacial acetic acid solution was added to the beaker with a rubber dropper. The chitosan aqueous dispersion was stirred with a magnetic stirrer for 3 h, and the supernatant was taken as the chitosan solution required for the experiment after standing for 1 h. PET Textiles (1 cm × 1 cm) were cut with scissors and ultrasonically cleaned in deionized water for 5 min to remove fiber fragments on surface. Then, the textiles were immersed in chitosan solution for 1 h and dried in a vacuum oven at 80 °C for 4 h. Then, the textiles were immersed in a beaker containing a mixed solution of PEDOT:PSS and SWCNTs for 1 h. Finally, it was placed in a vacuum drying oven and dried at 80 °C for 4 h to obtain PEDOT:PSS/SWCNTs/CS@PET textile.

### 2.3. Fabrication of PSCP Sensor

A layer of conductive silver paste was evenly coated on the upper and lower layers of the e-textile to ensure that the copper electrode and the e-textile could be closely contacted when subjected to external force. The upper and lower copper electrodes were tightly bonded to the fabric, and then the conductive silver paste was heated and cured. The outermost layer was insulated with tape to protect the electrode and reduce signal interference.

### 2.4. Characterization and Measurement

The morphologies and thicknesses of the PEDOT:PSS/SWCNTs/CS@PET textile were characterized by field emission scanning electron microscopy (SEM SUPRA-55, Oberkochen, Germany). The qualitative and quantitative analyses of the surface elements of samples were performed by energy-dispersive spectrometry (EDS, Ultim Max65, Oxford, UK) configured in the SEM equipment. The X-ray diffraction patterns (XRD) of PEDOT:PSS/SWCNTs/CS@PET textiles were determined by an automatic multi-purpose X-ray diffractometer (Rigaku Ultima IV, Tokyo, Japan). The sensor performance testing was carried out by means of a Keithley Instruments Model 2450 digital source meter (Tektronic, Beaverton, OR, USA).

## 3. Results and Discussion

### 3.1. Structural Framework of the Dual-Mode PSCP Sensor

**Figure 1:**
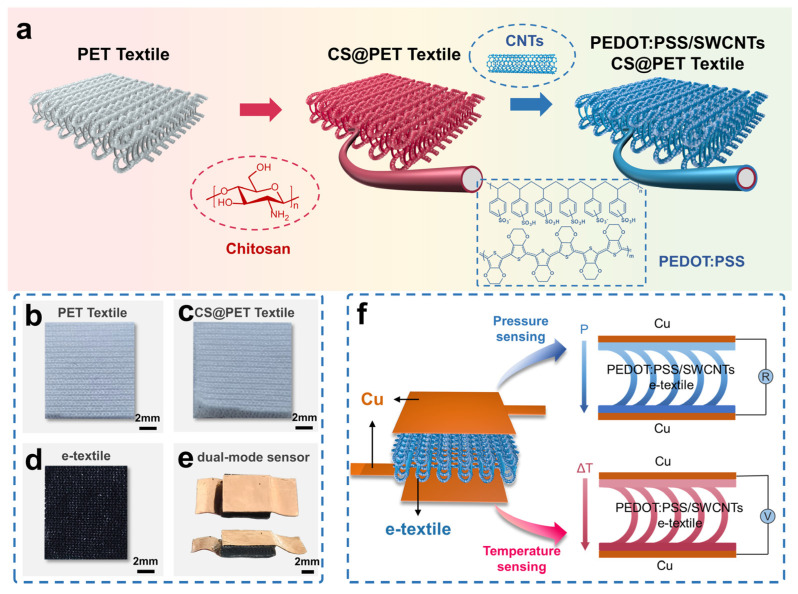
(**a**) Schematic of the preparation process of the PSCP sensor and the structure of the materials; (**b**) Initial textile; (**c**) CS@PET textile; (**d**) PEDOT:PSS/SWCNTs/CS@PET e−textile; (**e**) The physical drawing of the PSCP sensor; (**f**) Schematic structure of a dual-mode sensor for pressure and temperature.

Figure 1a demonstrates the fabrication process of textile sensors, specifically the modification of PEDOT:PSS/SWCNTs on elastic 3D spacer PET textiles by vacuum-assisted impregnation for efficient temperature–pressure sensing. PET textiles are polymer textiles prepared by large-scale textile technology, with outstanding cost-effectiveness, highly automated manufacturing, versatility, and biocompatibility. The prepared PET textile (as shown in Figure 1b) has super-elasticity and excellent durability, which are beneficial to the application of pressure sensing. At the same time, it also has good thermal insulation performance, which is beneficial for applying temperature sensing. To improve the interfacial interaction between PET textiles and PEDOT:PSS/SWCNTs, a thin CS adhesive layer was coated on PET textiles to form CS@PET textiles (as shown in Figure 1c). The presence of the chitosan layer ensures the water wettability of the textiles. Subsequently, CS@PET textiles were immersed in a PEDOT:PSS/SWCNTs mixed solution under vacuum assistance. During this period, due to the electrostatic attraction and hydrogen bonding between SWCNTs, PEDOT:PSS, and chitosan, conductive materials can spontaneously adhere and stably deposit on the surface of CS@PET textile fibers to form PEDOT:PSS/SWCNTs electronic textiles. Chitosan is a positively charged polyelectrolyte in solution with strong adsorption properties. And it contains polar groups such as hydroxyl and amino groups. When the cross-linking of PEDOT:PSS and CS occurs, a strong infrared absorption of chitosan associated with the N-H bending vibration is observed at 1560 cm^−1^ in the FTIR spectrogram. Hydrogen bonding or electrostatic interactions formed between chitosan and the host material usually explain this phenomenon [45,46]. The IR absorption band at 1190 cm^−1^ for the -SO_3_- molecule of PSS in chitosan and PEDOT:PSS is reshifted in chitosan-crosslinked PEDOT:PSS to 1180 cm^−1^. This can be attributed to the strong electrostatic interaction between PSS and chitosan [47]. Used in this study are carboxylated singlewalled carbon nanotubes with a large number of carboxylic acid groups introduced into the carbon nanotube structure. The interaction of the carboxylic groups on the surface of the carbon nanotubes with the amino groups on the surface of the chitosan polymer allows the carbon nanotubes to adhere more firmly to the surface of the PET textile [48]. The dual-mode sensor is fabricated using a typical packaging method, in which the conductive copper foil is attached to the upper and lower layers of the fabric surface of the PET textile with silver paste. Therefore, the sensor can simultaneously monitor the thermoelectric voltage caused by the temperature gradient and the resistance change caused by the external pressure.

### 3.2. Characterization of the Dual-Mode PSCP Sensor

**Figure 2:**
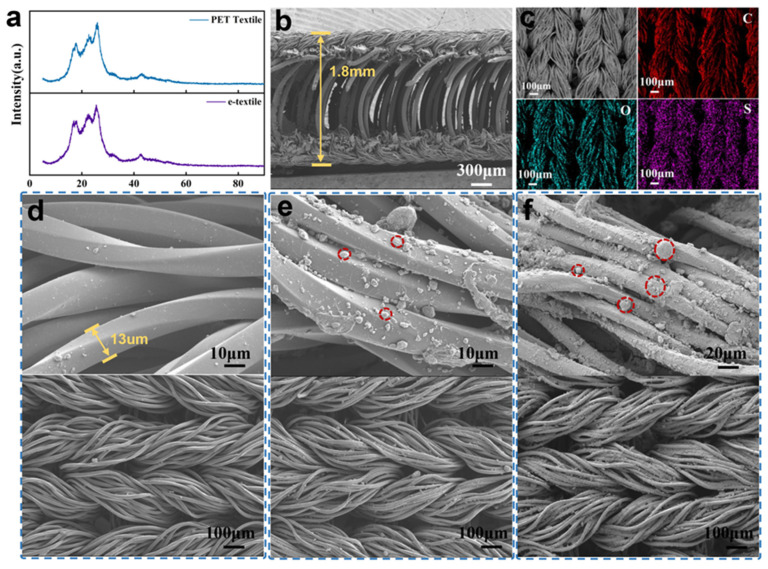
(**a**) XRD images of initial textile and conductive textile; (**b**) The side view of the conductive textile; (**c**) EDS mapping images of C.O.S. elements; (**d**) SEM image of the initial textile surface; (**e**) SEM image of the PEDOT:PSS/SWCNTs textile surface without chitosan; (**f**) SEM image of the PEDOT:PSS/SWCNTs/CS@PET textile surface.

Figure 2a shows the XRD characteristic peaks of the initial textile and the conductive textile (PEDOT:PSS/SWCNTs/CS@PET textile). It can be seen that the conductive material attached to the surface of the fibers affects the characteristic peaks. The morphology and microstructure of the original PET textile and conductive textile were observed by scanning electron microscopy (SEM). Figure 2b is the side view of the conductive textile. It can be seen that the upper and lower layers of the textile are connected by regular dense curved fibers. On the one hand, this provides sufficient space for textiles to deform under external pressure, which can improve the response range of the pressure sensor. On the other hand, the air interlayer in the middle can significantly reduce the efficiency of temperature conduction and improve the stability of the output thermal voltage of the temperature sensor. The energy-dispersive spectrometer (EDS, Ultim Max65, Oxford, UK) element mapping image (as shown in Figure 2c and Figure S1) shows that the C, O, S, and N characteristic elements in PEDOT:PSS/SWCNTs are evenly distributed on the surface of PET textiles, which proves that the conductive material is evenly attached to the surface of textiles. By comparing the SEM images of the initial textile and the e-textiles without/with chitosan, it can be found that the surface of the initial textile is smooth and has a twisted braided structure (as shown in Figure 2d). Even if there is conductive material adhesion on the surface of textiles without chitosan impregnation, they are irregular blocky protrusions and dispersed (as shown in Figure 2e). The conductive materials were uniformly adhered to the surface of the textiles after being impregnated with chitosan, especially the small conductive particles that were also adhered to the surface of the fibers (as shown in Figure 2f). CNTs remain in the form of large agglomerates. In the case of chitosan activation, the adhesion between conductive materials and textiles is enhanced due to surface electrostatic attraction and hydrogen bonding, which significantly improves the electrical properties of the conductive network. Figure S2 shows the compressive stress–strain curves of PET textile and PSCP sensor. As the compressive stress increases, compressive strain occurs in the PET textile and PSCP sensor. When the stress is released, the strain drops to zero, indicating that the shape is fully recovered and no plastic deformation exists. Cyclic compression tests were carried out on these two at 40% strain to demonstrate their fatigue resistance (Figure S2b,d). The stress–strain coincidence curves in Figure S2a,c show that the PET textile and PSCP sensor have good fatigue resistance with little energy dissipation during compression.

### 3.3. Pressure-Sensing Performance of the Dual-Mode PSCP Sensor

**Figure 3:**
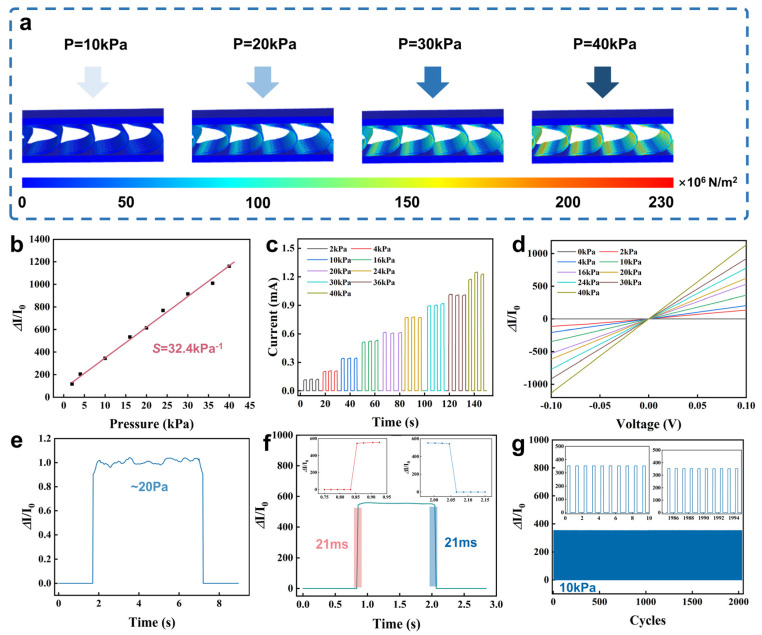
(**a**) The stress distribution of e−textiles inside the sensor under different pressures; (**b**) The sensitivity of the sensor; (**c**) The current response of the sensor is in the range of 0−40 kPa; (**d**) The voltage scanning results of the sensor in the range of −0.1 V−0.1 V; (**e**) The minimum pressure response of the sensor; (**f**) The response time and recovery time of the sensor to pressure; (**g**) The response current of the sensor over 2000 compression–release cycles. (I_0_: Initial current when no pressure is applied).

Due to the high elasticity, fatigue resistance, and stable conductive network, the PSCP sensor exhibits excellent pressure sensitivity. The finite element analysis is used to visualize the pressure-sensing process and clarify the sensing mechanism of the sensor. As shown in Figure 3a, the sensor deforms under external pressure, and the stress is concentrated at the junction and bending of the intermediate support fiber. More conductive paths are generated in the PEDOT:PSS/SWCNTs conductive network, resulting in a decrease in the resistance of the entire e-textile. Figure 3b shows the current variation of the sensor output current under different pressures, which has a good linear relationship. The sensitivity of the sensor is 32.4 kPa^−1^ in the pressure range of 0–40 kPa, which has obvious advantages over other textile-based sensors. Figure 3c shows the change of the output current during a continuous pressure-increasing process, which proves that the output current can correspond to the pressure values one by one. Figure 3d shows the voltage scanning of the sensor from −0.1 V to 0.1 V under different pressure loads, which proves that the e-textile maintains good ohmic contact with the copper electrode and the current change generated during the measurement process is only related to the resistance change of the e-textile. The minimum pressure that the sensor can measure is about 20 Pa (as shown in Figure 3e). The response time and recovery time of the sensor are about 21 ms (as shown in Figure 3f). Due to the twisting structure of the upper and lower layers of the textile, there is a twisting force between multiple fibers, which makes the conductive network of the textile able to quickly produce corresponding deformation when the pressure on the textile changes. This shows that, even at high compression frequencies, the sensor exhibits a fast reversible response to pressure stimuli and has good linear ohmic behavior, which are suitable for application scenarios such as touchscreens, encrypted transmission, etc. More importantly, Figure 3g exhibits stable pressure-sensing performance after 2000 cycles of repeated compression at a pressure of 10 kPa, confirming its long-term stability and durability in practical applications.

### 3.4. Temperature-Sensing Performance of the Dual-Mode PSCP Sensor

**Figure 4:**
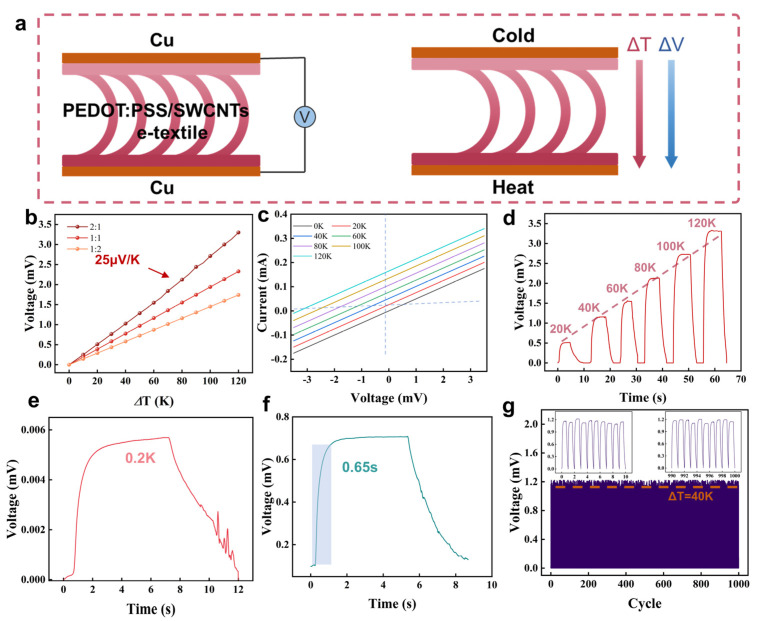
(**a**) Thermoelectric effect diagram of dual−mode sensor; (**b**) The Seebeck coefficient of PEDOT:PSS and SWCNTs after different proportions of impregnation; (**c**) I–V curves at different temperatures; (**d**) The thermal voltage generated by the dual-mode sensor at different temperatures; (**e**) The minimum detection limit of temperature difference; (**f**) Temperature response time; (**g**) Cyclic testing of temperature response.

As shown in Figure 4a, the thermal voltage is usually generated by the temperature difference between the upper and lower surfaces of the sensor. When a temperature difference is formed between the upper and lower surfaces of the sensor, the carrier migrates to the lower part of the temperature and generates a thermal voltage. As the temperature difference increases, more carrier migration will produce greater thermal voltage. Since PEDOT:PSS has excellent thermoelectric properties, the conductivity of the conductive network is further improved after doping with SWCNTs, and the 3D space textiles have good thermal insulation function. These advantages make the sensor have good temperature-sensing performance. Figure 4b shows the different mass ratios of PEDOT:PSS and SWCNTs. The sensors have excellent temperature-sensing performance and can maintain a stable Seebeck coefficient of 25 μV/K at a temperature difference of 0–120 K when the mass ratio is 1:2. The room temperature is 16 °C, and the temperature difference is set at 16 °C. As shown in Figure 4c, the curve intercept along the *X*-axis reflects the voltage generated by different temperature gradients. Figure 4d shows that the sensor can accurately respond to the external temperature range of 0–120 K. The temperature detection resolution of the sensor is crucial to ensure high-precision temperature sensing. The sensor can accurately detect a minimum temperature change of 0.2 K (as shown in Figure 4e). In addition, the low thermal conductivity of the sensor further ensures that a stable temperature difference can be quickly generated between the upper and lower layers, thereby achieving a temperature response of 0.65 s (as shown in Figure 4f). Figure 4g shows the repeated temperature change over 1000 cycles with a temperature difference of 40 K. The sensor exhibits a stable and repeatable output voltage response, excellent long-term durability, and reliability in practical applications.

### 3.5. Pressure- and Temperature-Sensing Performance of Dual-Mode Sensor

**Figure 5:**
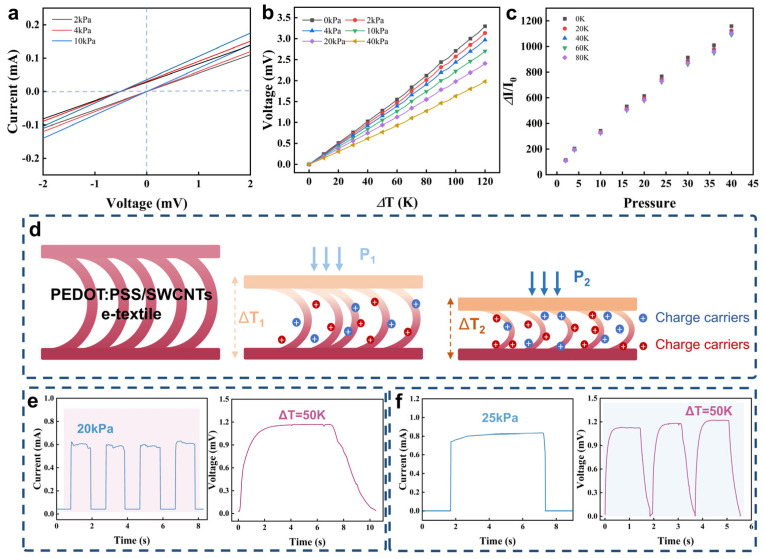
(**a**) I−V curves at different pressures and temperatures; (**b**) Seebeck coefficient under different pressures; (**c**) Pressure sensitivity at various temperature differences; (**d**) The sensing mechanism of pressure and temperature; (**e**) Multiple pressure tests at a temperature difference of 50 K; (**f**) Multiple temperature tests at 25 kPa. (I_0_: Initial current when no pressure is applied).

For dual-mode temperature–pressure sensing, the sensor should have a signal decoupling function to independently detect and distinguish temperature and pressure signals without interference. To study its two-parameter sensing ability, temperature and pressure stimuli are applied to the sensor at the same time. As shown in Figure 5a, when the pressure increases, the 3D conductive network of the textile will gradually compress as the pressure increases, resulting in a decrease in resistance and an increase in the slope of the current–voltage curve. The thermoelectric voltage generated by the temperature rise corresponds to the intercept of the current–voltage curve. According to this principle, the sensor can read these two independent parameters at the same time. As shown in Figure 5b, we recorded the relationship between the thermal voltage and the temperature difference in the range of 0–120 K under 0–40 kPa. It can be found that, in the low-pressure region, the slope of the curve changes little, indicating that the pressure has little effect on the temperature sensing, but, as the pressure increases, the slope of the curve decreases significantly. This is mainly because, under external pressure, the upper and lower layers of textiles are in close contact, and the air interlayer in the middle is compressed. The thermal conductivity between the two is significantly enhanced, which has a negative impact on the Seebeck coefficient. As shown in Figure 5c, the sensitivity curves of the sensor to pressure almost overlap under the temperature change of 0–80 K, which indicates that the temperature has little interference with the pressure response of the sensor. According to thermoelectric physics, the carriers inside the material will migrate from the high-temperature side to the low-temperature side under the temperature gradient, resulting in thermoelectric voltage (as shown in Figure 5d). The generated thermal voltage depends largely on the energy difference between the carriers on both sides of the material along the temperature gradient. It is also worth noting that the change in carrier energy with temperature can be reflected in the change in its energy band, while the pressure stimulation of 0–40 kPa has little effect on the energy band of the carrier. Therefore, when both temperature and pressure stimuli are applied, the thermal voltage depends mainly on the temperature gradient rather than the pressure. It is worth noting that the interference between temperature and pressure can be neglected in the low-pressure and low-temperature regions. Figure 5e shows that there is no effect on the response to temperature when the pressure changes at a low level. Figure 5f shows that there is little effect on the response to pressure when the temperature changes. Therefore, the signal decoupling mechanism of the sensor is mainly attributed to the pressure-independent energy band of the conductive material. This ensures that the voltage signal generated by the temperature stimulation and the resistance signal generated by the pressure stimulation will not interfere with each other, thereby achieving high-precision dual-mode detection without crosstalk.

The sensing properties of the PSCP sensor were compared with those in some other works, as shown in Table S1 [49,50,51,52]. The sensor showed superiority in pressure sensitivity, Seebeck coefficient, and response time.

### 3.6. Dual-Mode Sensor for Pulse and Body Temperature Measurement

**Figure 6:**
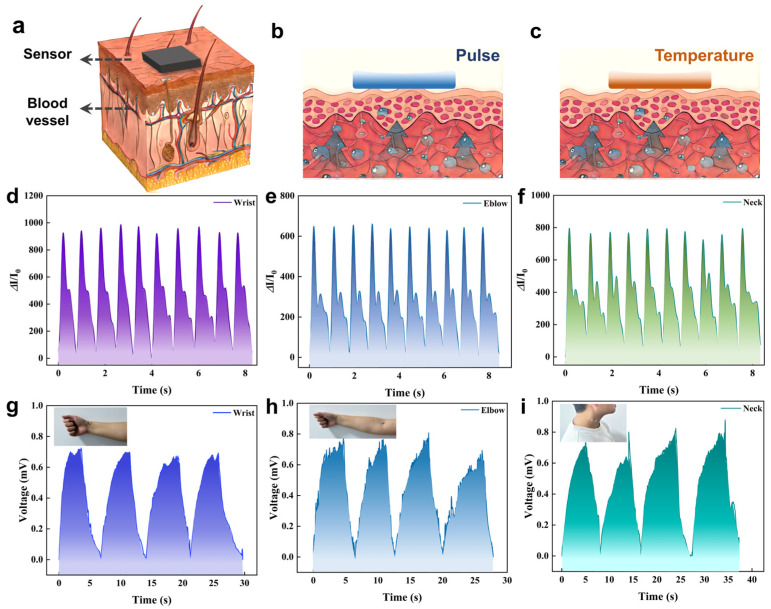
(**a**) A schematic diagram of the sensor measuring on the skin surface; (**b**) Pulse signal monitoring diagram; (**c**) Body temperature signal monitoring diagram; (**d**–**f**) Pulse signals in the wrist, elbow, and neck; (**g**–**i**) Temperature signals on the wrist, elbow, and neck. (I_0_: Initial current when no pressure is applied).

Based on the excellent sensing performance of the sensor, we used the sensor to measure the pulse signal and body temperature signal of a 25-year-old volunteer’s wrist, elbow, and neck. The schematic diagram of the sensor on the skin surface is shown in Figure 6a. When the sensor is placed above the arterial blood vessel, it is necessary to apply appropriate initial pressure to the sensor. When the blood vessel is deformed by external pressure, a larger pulse signal will be generated, which is convenient for signal acquisition (as shown in Figure 6b). At the same time, when the sensor is placed on the skin surface, the temperature on the side in contact with the skin will increase significantly. A temperature difference will be formed between the upper and lower layers. The human skin temperature is calculated by collecting the thermal voltage of the sensor (as shown in Figure 6c). As shown in Figure 6d–f, the sensor can detect pulse signals in the wrist, elbow, and neck, and it can be seen that the pulse rates of the three parts are the same, roughly 71 times per minute. Due to the differences in vascular elasticity, thickness, and blood flow velocity in different parts, the signal amplitude of the sensor is different. The body temperature signals collected synchronously by the sensor in three parts are shown in Figure 6h,i. Under the action of temperature difference, the thermal voltage excited by the sensor is stable at about 0.6 mV. According to the Seebeck coefficient calculation, for the sensor, the thermal voltage of 0.6 mV corresponds to a temperature difference of 24 K. Moreover, due to the different blood flow velocities in different parts, there are also some differences in the thermal voltage between the three. Among them, the blood flow velocity of the carotid artery is the fastest, and the corresponding skin surface temperature is also the highest. The brachial artery is slightly thinner, the blood flow velocity becomes slower, and the corresponding skin temperature decreases. The skin temperature of the thinnest radial artery was the lowest. It is worth noting that the skin of the wrist is the most flat and the contact between the sensor and the skin surface is the most suitable. Therefore, the collected pulse signal and body temperature signal are the most stable, and the noise influence is the smallest.

Despite these sensing properties, the prepared PSCP sensors have great potential for use in practical wearable applications. Due to the advantages of softness and breathability that the textile possesses, as shown in Figure S3a, the prepared PSCP sensor can fit well with the skin. To test its comfort and applicability, the sensor was worn on the subject’s wrist for five hours. After removing the sensors, no phenomena such as redness, swelling, or allergy were found on the skin at the wrist where the sensors were attached. The only marks on the surface of the skin at the test site were those caused by slight pressure from the sensor on the skin (shown in Figure S3b).

## 4. Conclusions

An integrated temperature and pressure dual-mode sensor was fabricated by mass-produced PET textiles attached to PEDOT:PSS and SWCNTs with high thermoelectric properties. The PSCP sensor possesses the ability to independently detect both temperature and pressure parameters with a Seebeck coefficient of 25 μV/K in the temperature detection range of 0–120 K and high sensitivity (32.4 kPa^−1^), fast response time (21 ms), and excellent cyclic stability (2000 times) in pressure sensing. Based on the different sensing mechanisms of the Seebeck effect and piezoresistive effect, the PSCP sensor realizes the decoupling of signals between the thermal voltage and piezoresistance, which is an urgent problem for multi-modal sensors. Meanwhile, based on the excellent temperature- and pressure-sensing performance, it realizes the independent and continuous detection of human pulse and body temperature signals. The PSCP sensors based on high-performance electronic textiles can independently detect both temperature and pressure parameters and have great potential in robot tactile sensing, wearable electronics, and medical monitoring devices.

## Data Availability

No primary research results, software or code have been included, and no new data were generated or analysed as part of our research.

## Supplementary Materials

The following supporting information can be downloaded at: https://www.mdpi.com/article/10.3390/mi16010092/s1, Table S1: The comparison of sensing performance with other sensors; Figure S1: EDS mapping images of N element; Figure S2: The compressive stress-strain curves of PET textile and PSCP sensor. (a) Compressive stress-strain curves for PET textile at different strains (20, 40 and 50%). (b) Compressive stress−strain curves for PET textile at 40% strain for 1000 cycles. (c) Compressive stress-strain curves for PSCP sensor at different strains (20, 40 and 50%). (d) Compressive stress−strain curves for PSCP sensor at 40% strain for 1000 cycles; Figure S3: (a) Physical diagram of the PSCP sensor fitting to the wrist. (b) Image of the skin on the surface of the wrist after five hours of wearing the PSCP sensor.
