# Supplementary material for: Dual-Mode Textile Sensor Based on PEDOT:PSS/SWCNTs Composites for Pressure–Temperature Detection
Source: Micromachines (Basel). 2025 Jan 14;16(1):92. doi: 10.3390/mi16010092 (PMC11767976; doi:10.3390/mi16010092)
Supplement: Supplementary file 1 [file micromachines-16-00092-s001.zip › micromachines-3361177-supplementary.pdf]

Dual-Mode Textile Sensor Based on PEDOT:PSS/SWCNTs Composites for Pressure–Temperature Detection

Ying Wang [1,*,#], Qingchao Zhang [2,#] and Zhidong Zhang [3,*]

[1] School of Energy and Power Engineering, North University of China, Taiyuan 030051, China
[2] School of Precision Instrument and Optoelectronics Engineering, Tianjin University, 92 Weijin Road, Tianjin 300072, China.
[3] School of Instrument and Electronics, North University of China, Taiyuan 030051, China;
* Correspondence: wangying1979@nuc.edu.cn (Y.W.); zdzhang@nuc.edu.cn (Z.Z.)
# These authors contributed to the work equally and should be regarded as co-first authors.

Sensing material	Pressure Sensitivity (kPa^{-1})	Pressure Response Time (ms)	Seebeck Coefficient (μV/K)	Temperature Response Time (s)	Ref
PEDOT:PSS@MS	0.411	42	9.42	1.2	[49]
PEDOT:PSS/CNT@ Melamine	-3.35%	134	35.9	>5	[50]
PEDOT:PSS/Spacer fabric	0.058	<80	25	<2	[51]
PEDOT:PSS/$Ti_3C_2T_x$	3.05	157	22.7	>10	[52]
PEDOT:PSS/SWCNTs/CS@PET Textile	32.4	21	25	0.65	Our work

Table S1. The comparison of sensing performance with other sensors.

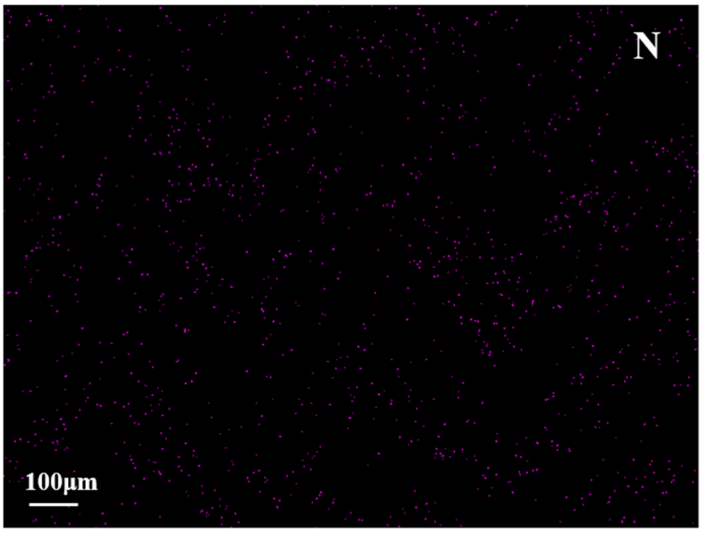

Figure S1. EDS mapping images of N element.

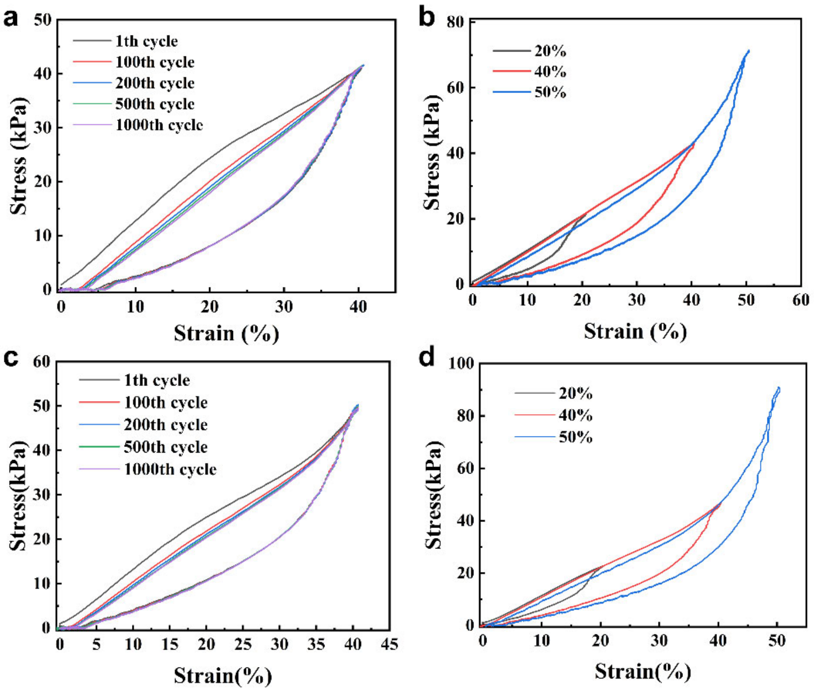

Figure S2. The compressive stress-strain curves of PET textile and PSCP sensor. (a) Compressive stress-strain curves for PET textile at different strains (20, 40 and 50%). (b) Compressive stress−strain curves for PET textile at 40% strain for 1000 cycles. (c) Compressive stress-strain curves for PSCP sensor at different strains (20, 40 and 50%). (d) Compressive stress−strain curves for PSCP sensor at 40% strain for 1000 cycles.

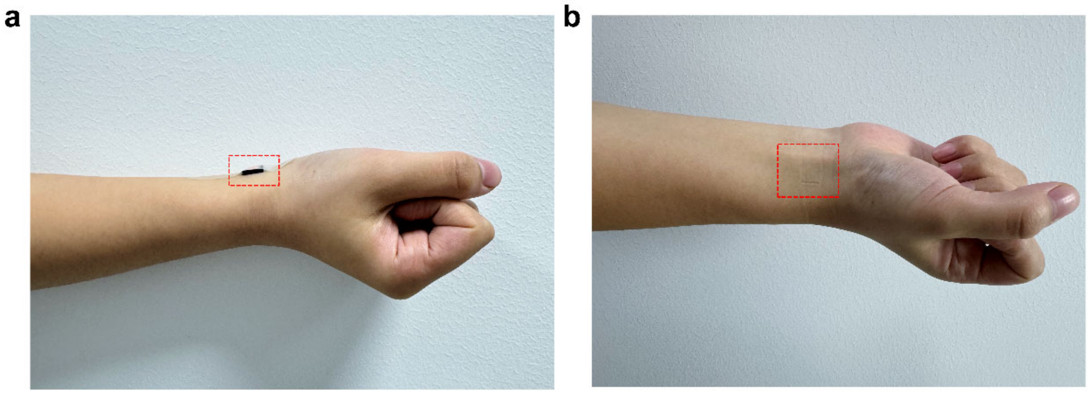

Figure S3. (a) Physical diagram of the PSCP sensor fitting to thes wrist. (b) Image of the skin on the surface of the wrist after five hours of wearing the PSCP sensor.
